# Hidden Harm: Detection of Abnormal Urinary Analysis in Alcohol and Polysubstance Abuse

**DOI:** 10.1192/bjo.2024.247

**Published:** 2024-08-01

**Authors:** Ghazanfar Shaikh

**Affiliations:** Royal College of Psychiatrists, London, United Kingdom

## Abstract

**Aims:**

This research study aims to identify the adverse effects of alcohol and polysubstance misuse on kidneys. The study also highlights the hidden harm caused by prescribed treatments such as PPI (Proton Pump Inhibitors) and other medications.

**Methods:**

The study was conducted in the summer of 2022 at an outpatient addiction treatment service. 63 patients (10% of the total prescribers), 49 males and 14 females participated in the study. All participants gave their consent, and data were collected including demographic details, substance misuse history, physical and mental health history, and prescribed treatments. We used a Combur-7 urinary dipstick to analyze the results provided in the kit.

**Results:**

Seven patients were not able to provide a sample. 60/63 patients' result showed abnormalities.

21 out of the 63 samples appeared dark and hazy. 7 samples were foul-smelling. 40 of the 63 patients were detected with a variable amount of leukocytes. 1 of the 63 patients was positive for nitrogen. The pH values range from 5 to 8. Specific gravity values were variable. 3/63 samples were positive for bilirubin. 58/63 samples were positive for protein. 19/63 samples detected variable amounts of red blood cells. 5/63 samples detected for ketones and glucose were negative in all samples.

**Conclusion:**

Long-term alcohol abuse can compromise the ability to manage fluid volume and electrolyte balance. Extreme serious abuse can also impact acid-base balance, homeostasis, and even hormonal control regulated by the kidneys could be affected. This situation further complicates the presence of liver disease.

Cocaine abuse can cause acute kidney injury (AKI), malignant hypertension, and vasculitis and can lead to chronic kidney disease (CKD). Heroin-associated nephropathy (HAN) can lead to nephrotic syndrome and could progress to end-stage renal failure.

Tobacco, solvents, amphetamines, and ecstasy can aggravate a wide range of kidney diseases by their direct or indirect effect on kidney functions.

Long-term use of proton pump inhibitor and other medications such as NSAID, pregabalin, and diuretics, may affect kidney functions.

The Opiate substitute treatment dose needs to be adjusted in the presence of poor kidney functions to reduce morbidity and mortality. Early screening is required for all patients on long-term OST and other medications for comorbid illnesses.

